# Macrophage proliferation, provenance, and plasticity in macroparasite infection

**DOI:** 10.1111/imr.12221

**Published:** 2014-10-15

**Authors:** Dominik Rückerl, Judith E Allen

**Affiliations:** Institute for Immunology and Infection Research, School of Biological Sciences, University of EdinburghEdinburgh, UK

**Keywords:** IL-4 receptor, monocyte, macrophage, wound repair, inflammation, helminth infection

## Abstract

Macrophages have long been center stage in the host response to microbial infection, but only in the past 10–15 years has there been a growing appreciation for their role in helminth infection and the associated type 2 response. Through the actions of the IL-4 receptor α (IL-4Rα), type 2 cytokines result in the accumulation of macrophages with a distinctive activation phenotype. Although our knowledge of IL-4Rα-induced genes is growing rapidly, the specific functions of these macrophages have yet to be established in most disease settings. Understanding the interplay between IL-4Rα-activated macrophages and the other cellular players is confounded by the enormous transcriptional heterogeneity within the macrophage population and by their highly plastic nature. Another level of complexity is added by the new knowledge that tissue macrophages can be derived either from a resident prenatal population or from blood monocyte recruitment and that IL-4 can increase macrophage numbers through proliferative expansion. Here, we review current knowledge on the contribution of macrophages to helminth killing and wound repair, with specific attention paid to distinct cellular origins and plasticity potential.

This article is part of a series of reviews covering Monocytes and Macrophages appearing in Volume 262 of *Immunological Reviews*.

## Introduction

Infection with multicellular parasites represents one of the most significant challenges to both veterinary and public health. Medically relevant multicellular parasites are called ‘helminths’, a term that does not accurately reflect the vast phylogenetic diversity of this group, which includes the platyhelminths (flukes and tapeworms) and nematodes (roundworms). These animal phyla diverged over 1 billion years ago, predating the split between vertebrates and invertebrates 1. Despite the phylogenetic distance between them, helminths as well as many insects induce a characteristic immune response that at its core, features the production of interleukin-4 (IL-4) and IL-13 by T-helper 2 (Th2) cells and a range of innate cells. Thus, we are apparently hardwired to mount a type 2 response on exposure to multicellular parasites 2.

In humans, helminths predominantly cause disease in developing countries but are a major veterinary problem throughout the world and often establish long-lasting infections that can last years and even decades. As a consequence, these infections are rarely lethal but can have devastating effects on life quality 3,4. Often, however, infections are asymptomatic or associated with less obvious repercussions (e.g. growth retardation, blunted mental development, etc.) caused by nutritional restrictions imposed on the host 5. To maintain fitness, the infected individual must cope with large motile foreign organisms that cannot easily be digested or removed, and for which an aggressive host response would be self-damaging. Further, these parasites often directly damage host tissue during migration or feeding. The host must thus strike a balance between immune suppression to minimize self-harm, the repair of damaged tissue and a sufficiently robust anti-worm response to keep parasite numbers below a level that compromises fitness. Type 2 immunity appears to be the response mammals have evolved to cope with this challenge. Th2 cells that produce IL-4, IL-5, IL-9, IL-10, and IL-13 and B cells producing immunoglobulin E (IgE) are the central players in the adaptive type 2 response and the innate cells associated with type 2 cytokines include eosinophils, mast cells, basophils, macrophages, and type 2 innate lymphoid cells (ILC2). Together, these pathways promote parasite killing and expulsion but are also key components of tissue repair pathways that are fundamentally non-inflammatory 2,6,7.

In this review, we focus on the role of macrophages in keeping the host fit in the context of a type 2 response induced by helminth infection. Macrophages have long been center stage as the target of Th1-type responses with a clear role in containment of microbial pathogens, particularly intracellular bacteria and protozoa. The function of macrophages activated by Th2 cytokines has been far more difficult to establish, despite their abundance in almost every helminth infection as well as other type 2 conditions such as allergy and asthma. While IL-4Rα ligation by either IL-4 or IL-13 has a dramatic effect on the macrophage transcriptional landscape 8,9, the specific functions of macrophages activated via the IL-4Rα have yet to be fully defined. Here, we use the term alternatively activated macrophage (ΑΑΜΦ) to define macrophage phenotypes that rely on the IL-4Rα 10. However, even when considering activation via a single receptor there is a broad heterogeneity of context-dependent macrophage phenotypes observable. This makes it difficult to identify unifying functional properties of ΑΑΜΦ. With an emphasis on our own research, we highlight here the knowledge that has been gained from studying macrophage activation in helminth models, which has provided broad insight into macrophage biology.

## Macrophage proliferation

We have been using a murine model of filariasis, *Litomosoides sigmodontis*, to understand the mechanisms of resistance and susceptibility to filarial nematode infection. Infective larvae (L3) invade via the skin, migrate through the lymphatics, and enter the pleural cavity, where they mature to adulthood. In susceptible BALB/c mice, the adult worms produce blood-circulating offspring (microfilariae) by day 50 postinfection. In resistant C57BL/6 mice the parasites are killed in the pleural cavity prior to reaching sexual maturity. This has proved a powerful model for the study of filariasis 11 and highlighted a critical role for T-regulatory cells in the susceptible phenotype of BALB/c mice 12. To investigate the contribution of macrophages to parasite killing in the resistant C57BL/6 strain, we depleted blood monocytes to prevent their recruitment to the infection site. To our surprise, we saw no reduction in the high macrophage numbers accumulating in the pleural cavity of infected mice. Time course assessment over the first 10 days of infection demonstrated that although macrophages were steadily increasing in number, their flow cytometry profile remained essentially the same as in naive mice, with no evidence of monocyte or neutrophil infiltration 13. This suggested that the resident macrophage population was expanding by other means than recruitment from the blood. Indeed, using both Ki67 staining and a 3 h BrdU pulse, extensive proliferation of the resident F4/80^hi^ population was observed 13.

Macrophage expansion and survival had previously been associated with CSF1R signaling and differentiation of monocyte derived macrophages 14–17. However, because helminth-driven macrophage proliferation was independent from blood monocyte recruitment and the numbers of cells accumulating at the site of infection were significantly reduced in IL-4^−/−^ mice 13, we tested the possibility that IL-4 was directly responsible. An injection of IL-4 complex (IL-4C) [a mixture of IL-4 with anti-IL-4 for increased bioactivity 18] into the peritoneal cavity led to dramatic macrophage proliferation 13. Remarkably, proliferative expansion was not restricted to the serous cavities but was observed in the liver 13, spleen, and lungs (authors’ unpublished observation). This was consistent with previously published data showing that IL-4 delivery, either in complex form or by mini-osmotic pump, led to macrophage hyperplasia in the liver, spleen and bone marrow 18. In both the Milner *et al*. study and ours 13,18–20, macrophage expansion following IL-4C delivery was independent of the adaptive immune system, occurring in RAG^−/−^ mice but not in Stat-6^−/−^ or IL-4Rα^−/−^ animals.

IL-4C injection allowed us to take a reductionist approach to address the factors that contribute to helminth-driven macrophage proliferation. Using macrophage-intrinsic competitive bone marrow (BM) chimeras in which Ly5.1^+^ WT mice are reconstituted with a 1:1 mix of Ly5.1^+^ WT and IL-4Rα^−/−^ Ly5.2^+^ BM, we established that IL-4 acts directly on the macrophages and IL-4Rα expression is essential to initiate a program of proliferation 19. However, important differences between the IL-4C model and *L. sigmodontis* infection were observed using these 50:50 WT:IL-4Rα^−/−^ chimeras. No BrdU incorporation into IL-4Rα^neg^ cells was observed following IL-4C injection, but during the early stages of *L. sigmodontis* infection, IL-4Rα^neg^ cells did incorporate BrdU, albeit at significantly lower level than the WT cells. As discussed below, we subsequently identified CSF1-dependent mechanisms to be responsible for the residual proliferative expansion of these cells during infection. Critically, IL-4Rα positive macrophages have a competitive advantage, proliferating to a greater extent and eventually outnumbering their IL-4Rα negative counterparts 19. Further, both macrophage proliferation and numbers are reduced in IL-4^−/−^ and IL-4Rα^−/−^ mice in all infection models tested, emphasizing the central role of IL-4 in driving macrophage accumulation during infection. Because IL-13 can also signal through the IL-4Rα we addressed whether IL-13 was also capable of inducing macrophage proliferation. We generated an IL-13 complex and demonstrated that it was equivalently able to drive macrophage proliferation when delivered intraperitoneally 19. However, a role for IL-13-mediated proliferation in a physiological setting has yet to be established.

The source of IL-4 (or IL-13) that drives macrophage proliferation during helminth infection is likely to vary with tissue and stage of infection 6 and may include mast cells, eosinophils, or innate lymphoid cells among others. However, our data suggest that during infection, CD4^+^ lymphocytes are required to generate significant macrophage numbers. In the absence of either RAG genes or class II, there is no expansion or alternative activation of macrophages in a peritoneal implant model of filarial infection 21. In the *L. sigmodontis* infection model, proliferation does not occur until the onset of the adaptive immune response and does not occur in infected RAG-deficient mice 13,19. Along with the need for high concentrations of IL-4 19, these data strongly suggest a requirement for cognate T-helper interactions to initiate macrophage proliferation in the infection context. Although macrophage accumulation in both IL-4C and infection models is the direct result of IL-4 driven proliferation, anti-apoptotic properties of IL-4 may also make a significant contribution to the final numbers.

## Macrophage provenance

Our discovery that IL-4 could drive expansion of resident macrophages occurred as a new paradigm in macrophage biology was unfolding. The established dogma had been that tissue-resident macrophages were derived from circulating bone marrow-derived monocytes. With new fate mapping technologies it became apparent that in most tissues including the serous cavities 22,23 the resident macrophages are established prenatally and are retained throughout the life of the animal through proliferative self-renewal (reviewed in 24,25). The notable exceptions to this rule are macrophages in the skin and GI tract 26,27.

**Fig 1:**
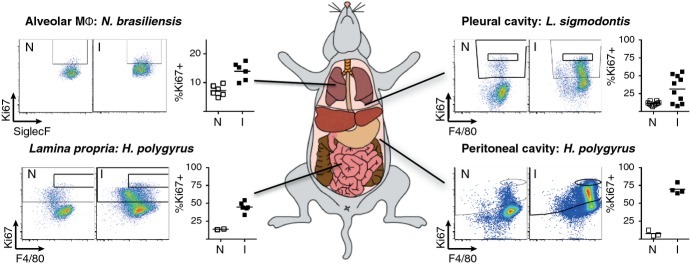
Proliferative expansion of macrophages (MΦ) is induced in various helminth infection models. Representative dotplots of one naive (N) and one infected (I) animal shown. Graphs indicate percent of F4/80^+^ macrophages that are Ki67^+^. Data points depict individual animals. Detailed description of materials and methods used in similar experiments can be found in Jenkins *et al*. 19. Data from *N. brasiliensis* infected alveolar macrophages isolated from whole tissue digests (CD11b^low^ SiglecF^+^ F4/80^+^ CD11c^high^) kindly provided by Dr. Tara E. Sutherland.

To verify the source of proliferating macrophages in our *L. sigmodontis* infection model, we used tissue-protected BM chimeras 13 in which resident macrophages in the body cavities are protected from radiation damage and thus not replaced by BM-derived cells. Using the recipient/donor ratio found in the blood versus the body cavity of naive animals, it is possible to determine whether the macrophages in infected tissues are of BM origin or not. Using this method, we were able to show that the expanding macrophage population following both IL-4C injection and *L. sigmodontis* infection were derived from tissue-resident cells 13. The tissue-protected BM chimeras thus allowed us to formally demonstrate that the expanding cells were not derived from circulating blood monocytes 13. However, AAMΦ in the GI tract of nematode infected mice are largely monocyte-derived 28, and we have observed proliferation of macrophages in the *lamina propria* of mice infected with the GI nematode, *Heligmosomoides polygyrus* (*Fig. *1). We thus investigated whether IL-4-driven proliferative expansion was restricted to tissue-resident macrophages or if IL-4 could act similarly on monocyte-derived cells. Using the tissue-protected chimeras, we demonstrated that injection of thioglycollate together with IL-4C stimulates the proliferation of recruited bone marrow-derived monocytes, while IL-4C alone stimulates resident cell expansion. Thus the ability of IL-4 to stimulate proliferation is not restricted to resident cells 13,20.

Macrophage self-renewal in most tissues relies on the CSF-1 receptor (CSF1R) 24, and CSF-1 has been shown to be required for both the steady state maintenance of resident peritoneal macrophages as well as recovery of the resident population by proliferative expansion following an inflammatory response 29. In our experiments with 50:50 WT:IL-4Rα^−/−^ bone marrow chimeras, steady-state proliferation in PBS-treated animals was not influenced by IL-4Rα signaling 19, confirming that IL-4 is not needed for local self-renewal of peritoneal macrophages 30. We hypothesized that instead, IL-4 functioned by enhancing steady state proliferation, perhaps by CSF1R upregulation or increased ligand production. To test this, the CSF1R was blocked during IL-4C delivery. IL-4-induced proliferation was undiminished in the presence of the CSF1R antibody demonstrating complete independence from the CSF1R 19. However, CSF1R blockade during *L. sigmodontis* infection suggested that CSF−1 does contribute to macrophage proliferation early in infection but is superseded by IL−4 presumably upon entry of Th2 cells. Supporting this, CSF1R blockade at day 8 postinfection inhibited macrophage proliferation only in the animals that did not yet exhibit evidence of high IL−4 exposure 19. Thus, IL-4 rather than acting via the CSF1R appears to unleash macrophages from their dependence on the CSF1R for proliferation.

IL-4 has the capacity to induce alternative activation as well as proliferation in both resident and recruited settings 13. This raised the fundamental question of whether IL-4Rα-activated macrophages of distinct origin are functionally similar. To address this P'ng Loke and colleagues 20 compared the transcriptome of resident versus recruited macrophages exposed to IL-4 using delivery of IL-4 into naive or thioglycollate-injected mice. The results dramatically illustrated that cellular origins impart a far larger stamp on the transcriptome than IL-4 exposure. They further demonstrated that many of the markers traditionally associated with alternative activation were only present on the blood monocyte derived population, including the mannose receptor, Raldh2 and PD-L2. The difference in Raldh2 expression translated into the ability of only monocyte derived cells to promote the differentiation of FoxP3 cells from naive CD4^+^ cells via retinoic acid production. In contrast, some IL-4-induced gene products, such as Uncoupling protein 1, were seen only in the resident derived cells. These phenotypic differences between cells of embryonic origin versus bone marrow origin were verified in *L. sigmodontis* versus *Schistosoma mansoni* infections, respectively 20. Thus, macrophage provenance is of critical importance in understanding the outcome of a type 2 immune response.

**Fig 2:**
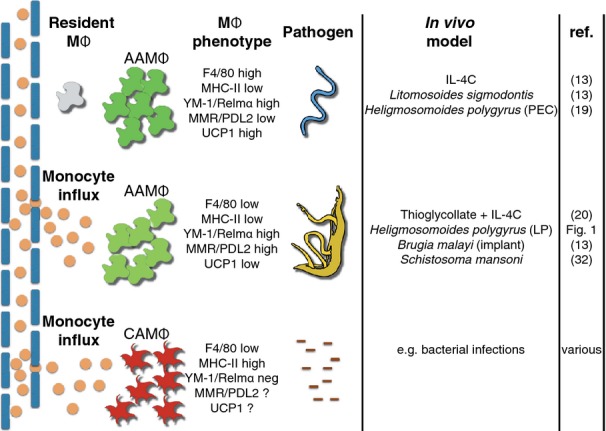
*In vivo* recruitment/expansion of macrophages (MΦ) is dependent on the infectious agent. Macrophage numbers can expand in the tissues either due to recruitment, proliferation, or a combination of both. This figure illustrates different infection models or experimental manipulations that can lead to these distinct outcomes.

In addition to the expansion of resident pleural macrophages in the *L. sigmodontis* model, we also observed that infection with the purely gastrointestinal nematode, *H. polygyrus*, caused expansion of peritoneal resident cells. This was somewhat surprising, as the parasite does not enter the peritoneal space. However, previous studies have shown the presence of Th2 cells in the peritoneal cavity of infected mice 31, consistent with our prediction that T-helper cells are instrumental in inducing proliferation. In both the *L. sigmodontis* and *H. polygyrus* models, shielded bone marrow chimeras were used to verify the resident origin of the pleural and peritoneal macrophages, respectively. However, these infections may prove to be exceptions to the rule and in many, if not most, Th2 infections, there is likely to be recruitment of monocytes from the blood. Because the importance of macrophage origins has only recently become recognized, little information is available in different model systems, and thus we are relatively ignorant of what determines when a recruited population is dominant over resident expansion. Even in the *L. sigmodontis* model, over time there is increasing numbers of recruited monocytes (unpublished observation). We presume that ‘non-inflammatory’ entry of the infective larvae combined with local induction of IL-4 by innate cells means blood cell recruitment is initially avoided. It may be that only subsequent parasite death is sufficient to trigger monocyte recruitment. In settings in which bacteria are present, such as infection of the gastro-intestinal tract, monocyte recruitment will likely dominate 28 (*Fig. *2).

Two important papers have recently addressed macrophage origins in the liver of *S. mansoni* infected mice 32,33. During patent *S. mansoni* infection, eggs become trapped in the liver and these induce a host-protective Th2-mediated granuloma, in which ΑΑΜΦ are a major component. Using intra-vital imaging and cell transfer experiments with Cx3Cr1^GPF/+^ mice, Girgis *et al*. 33 demonstrated that ΑΑΜΦ in the liver granulomas arise predominantly from blood monocytes. Similarly, Nascimento *et al*. 32 using careful phenotyping of the developing granuloma and pulse chase experiments demonstrate that the majority of macrophages are bone marrow derived. Importantly, Nascimento *et al*. 32 show that there is robust infection-induced proliferation of the resident macrophages, but this does not translate into significant cellular expansion. The dominance of the recruited monocyte population may reflect an inflammatory environment caused by leakage of intestinal bacteria into the portal bloodstream that may occur as eggs successfully cross into the gut lumen 32. Indeed, the authors show that the macrophage phenotype is highly heterogeneous, with clear evidence of alternative activation but also the production of iNOS and TNF. We have observed macrophage proliferation in the serous cavities, *lamina propria*, and the lung in different Th2-inducing models (*Fig. *1). Nascimento *et al*. 32 extend these observations to the liver, but illustrate that the dynamics by which macrophages accumulate are highly complex. Importantly, even when proliferation is not observed, this may be due to the narrow time window in which proliferation can be seen 19. Thus, even when it is straightforward to assess the tissue-resident versus BM origin of macrophages in inflamed tissue, determining whether the final numbers are the result of proliferation, recruitment, or enhanced survival will still be challenging.

With the new understanding of macrophage origins and proliferation capacity, the functional potential of macrophages activated in type 2 immunity gains even more complexity. However, at a more fundamental level we are still remarkably ignorant of the specific contribution of IL-4Rα activated macrophages to disease outcome in most type 2 disease settings. In the following sections, we discuss what is currently known about the specific functions of AAMΦ but also new leads from transcriptional analysis comparing WT versus IL-4Rα^−/−^ macrophages 9.

## Helminth killing

Immune-mediated killing of parasitic worms has been repeatedly demonstrated in animal models, and, with rare exceptions, type 2 immunity is required. In the case of several gastrointestinal models, delivery of IL-4 and/or IL-13 alone triggers mucus production, smooth muscle contraction and epithelial turnover, which is sufficient to mediate expulsion from the gut 34. More broadly IL-4 or IL-4 receptor deficiency confers susceptibility on resistant murine strains for a range of helminth infections 6. Helminth infected humans are reported to display a ‘modified Th2’ response that results from sophisticated adaptations employed by the parasites to divert the host immune reaction as well as host adaptations to avoid immune-mediated damage 35–37. Together these allow chronic infections to establish and can make deciphering the appropriate protective response difficult. This is further complicated by the dynamics of helminth life cycles and life histories such that the killing mechanisms depend on multiple factors that include the developmental stage of the parasite as well as the tissue through which the parasite migrates and eventually resides.

This complexity is exemplified by the model filarial nematode, *L. sigmodontis*. In primary infection, over half of the infective-stage L3 larvae are killed in the skin immediately after infection or during their migration to the pleural cavity 38. Once the remaining larvae make it to the pleural cavity and start to undergo the molting process into L4 stage larvae and adult worms, further killing is relatively slow. Furthermore, the mechanisms by which the larvae and adults are killed by the immune system differ 39. In susceptible but immunocompetent mouse strains (e.g. BALB/c) this process can take several months (>90 days) and even in resistant strains (e.g. C57BL/6) the parasites survive for several weeks (approximately 40–50 days) 38. Similarly, on secondary infection with the gastrointestinal parasite *H. polygyrus* larvae are killed, but adult parasites implanted directly into the gut lumen of the immune recipient can establish patent infection 40. In general, early developmental stages are more vulnerable to immune attack than later stages, perhaps because of the superior ability of parasites that survive to adulthood to modulate the host response 41. Some parasites create their own ‘immunoprivileged’ sites by forming cysts (e.g. *Echinococcus granulosus*) which show a high degree of immunomodulatory activity diverting potentially protective immune responses 42. Thus, one aim of the immune response is to trap parasites during their migration/development before a successful immune evasion strategy is in place. This appears to be the basis of most successful model vaccines 43. For example, *L. sigmodontis* larvae exhibit limited migratory capacity in vaccinated mice 44, and *H. polygyrus* larvae become trapped inside the gut wall in resistant or immune mice 45,46. A range of type 2 cytokines, cell types, and antibodies have been implicated in protective immunity in many different helminth models, but only recently have macrophages been receiving due attention 47.

The specific contribution of macrophages and, moreover, IL-4Rα-dependent activation in the killing and expulsion of helminths is still unclear. Macrophages make up a large proportion of the cells present at the site of various helminth infections 48 and are a major constituent of granulomas forming around parasites 49–51. Macrophage depletion using clodronate liposomes has demonstrated their importance for expulsion of intestinal parasites like *Nippostrongylus brasiliensis* or *H. polygyrus* 28,52. Similarly interference with macrophage function through injection of carrageenan or carbon particles enhances the survival of *Brugia malayi* and *B*. *pahangi* larvae in the peritoneal cavity of infected animals 53,54. Of particular interest is recent data suggesting that while macrophages are important in larval killing, they don't act alone. Bonne-Année *et al*. 55,56 demonstrated that *in vitro* macrophages and neutrophils work together to kill *Strongyloides stercoralis* larvae.

The evidence that macrophages are involved in worm killing is strong, but whether this requires alternative activation or is possibly hampered by it is still contentious. In a very recent study, expression of alternative activation markers positively correlated with resistance to infection with *H. polygyrus* and depletion of macrophages using clodronate liposomes resulted in reduced resistance 51. However, the study did not directly assess the role of the IL-4Rα, and it is possible that although macrophages themselves might be important for efficient killing of invading parasites the IL-4Rα-mediated activation might not. Most of the studies attempting to resolve the specific role of AAMΦ have used mice with LysM-Cre mediated deletion of the floxed IL-4Rα gene, which is relatively inefficient particularly on peripheral blood monocytes 57. Additionally, even if IL-4Rα-induced macrophage genes contribute to worm killing, these may be countered by proteins that inhibit Th2-induced effector pathways. For example, Arginase and RELMα, products strongly associated with alternative macrophage activation, can act as negative feedback regulators directly limiting the Th2 response 58,59. Thus, it may not be surprising that efficient elimination of the gastrointestinal nematodes *N. brasiliensis* or *Trichinella spiralis* was unimpaired in LysMCre IL-4Rα^flox/−^ mice relative to WT 60–62. Critically, a role (or lack thereof) for AAMΦ in one infection system may not apply to all nematode infections as the Th2 effector mechanisms involved can be quite distinct 34,63.

**Fig 3:**
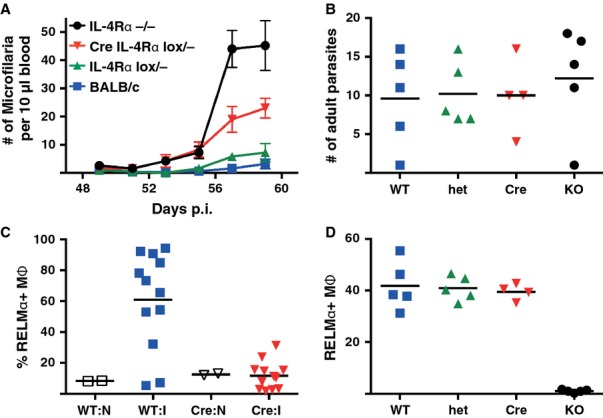
Expression of IL-4Rα by myeloid cells is required for optimal immune responses to filarial infection. BALB/c (blue squares; WT), IL-4Rα-deficient (black circles; KO), IL-4Rα heterozygous (green upward triangles; het), or LysMCre IL-4Rα ^flox/−^ (red downward triangles; Cre) were infected by subcutaneous injection of 25 *L. sigmodontis* L3 stage larvae. (A) At the indicated time points, 10 μl venous blood were collected from each mouse and the number of microfilaria present assessed by microscopic analysis. Data points indicate mean and SEM of 4–5 mice per group. (B) Number of adult parasites present in the pleural cavity of the mice analyzed in (A), 60 days after infection. Data points indicate individual animals and lines indicate mean. (C) Percent Relm-α expressing macrophages 10 days postinfection in naive (N) or infected (I) BALB/c (WT) or LysMCre IL-4Rα ^flox/−^ (Cre) mice. (D) Percent Relm-α expressing macrophages in the mice analyzed in A, 60 days postinfection. Detailed description of materials and methods used in similar experiments can be found in van der Werf *et al*. 36 (A, B) and in Jenkins *et al*. 13 (C, D).

Data from our laboratory using the filarial *L. sigmodontis* model suggest that there is a role for IL-4Rα-mediated macrophage activation in the control of this tissue nematode infection but also revealed a key difficulty with the LysMCre mice in the context of IL-4Rα expression. Infected LysMCre IL-4Rα^flox/−^ mice showed increased number of circulating microfilaria at late stages of infection (*Fig. *3) indicating a role for AAMΦ in either killing the circulating microfilaria or impairing female fertility. To our surprise, standard markers of alternative activation (i.e. RELMα and YM1) in the macrophage specific KO while absent as expected at day 10 were no different to wildtype animals at the late stage of infection (d60). Because LysMCre-mediated deletion of the floxed IL-4Rα gene is relatively inefficient 57 we predicted that chronic IL-4 exposure was causing an outgrowth of IL-4Rα sufficient macrophages, such that by day 60 post infection all macrophages were wildtype. This was consistent with our data utilizing 50:50 WT:IL-4Rα^−/−^ chimeras injected with IL-4, in which the WT macrophages outcompete their gene deficient counterparts 19. The competitive advantage during nematode infection was confirmed with *H. polygyrus*, in which the proportion of RELMα positive peritoneal macrophages in *LysMCre IL-4Rα*^*flox/−*^ mice increased from 4% in naive mice to 25% at day 14 and 70% by day 28, by which point >50% of the macrophages expressed the IL-4Rα 19.

Because actual IL-4Rα deletion occurred only early in infection, the data suggest AAMΦ act at early stages of infection to influence later resistance. That early events can determine the later outcome of infection is consistent with data in which T-regulatory cell depletion just prior to *L. sigmodontis* infection dramatically decreases microfilaria numbers at day 60 64. This hypothesis would also be in line with data showing that host immune status at the time of infection influences parasite developmental rate and subsequent fecundity 65. The failure of macrophages to efficiently alternatively activate early in infection could therefore lead to an altered parasite developmental program which allows the parasite to produce more offspring. One caveat is that LysMCre will also mediate IL-4Rα deletion in granulocytes, and thus we cannot be certain that the impaired microfilarial killing is specifically due to macrophages.

A recent report by Esser-von Bieren *et al*. 46 sheds new light on the potential contribution of macrophages to nematode killing. Using mice that lack antibodies (JH^−/−^) or activating Fc receptors (FcRγ^−/−^), they demonstrate in a model of secondary *H. polygyrus* infection that antibodies activate macrophages to trap and immobilize infective larvae. Arginase was important for killing as discussed below. In this setting, the ability of antibodies to induce arginase was independent of the IL-4Rα, but in its absence macrophages fail to accumulate at the infection site 46. Thus, even if the IL-4Rα is not always needed to induce macrophage effector molecules, it may be essential to expand macrophages in sufficient numbers to perform non-IL-4Rα-dependent functions.

It is therefore still early in our understanding of the mechanisms by which AAMΦ may contribute to parasite control. In the sections below, we discuss some of the findings about well-known markers of alternative activation and their role in resistance to helminth infection as well as more recent data from our laboratory identifying potential new effector molecules not previously associated with AAMΦ. Due to the fact that parasites can survive for very long periods of time in their respective hosts, it is reasonable to expect that a range and combination of different effector mechanisms need to work in concert to achieve effective parasite expulsion or killing. Of note, several AAMΦ products have been associated with an immune modulatory function rather than direct anti-parasitic effector responses. Thus, in many circumstances AAMΦ may not have a primary role in parasite killing but act to contain excessive inflammation and maintain tissue homeostasis.

## Effector molecules and pathways

### Chitinase-like proteins

YM1 [chitinase-3-like-3 (Chi3l3)] is one of the most highly expressed genes in alternatively activated macrophages 8,9,66. Upon activation with IL-4, macrophages accumulate large amounts of intracellular YM1 that can be readily detected by flow cytometry even without the use of Brefeldin A or Monensin and is simultaneously released in high quantities into the surrounding tissue 13,67. YM1 is part of the glycoside hydrolase family 18, which includes enzymatically active, chitin-degrading enzymes (i.e. chitotriosidase and AMCase) as well as enzymatically inactive, chitinase-like proteins (CLPs), e.g. YM1, BRP39, YM2 (reviewed in 68). Whereas the chitin-degrading enzymes are highly conserved across the animal kingdom, the CLPs represent relatively recent gene duplication events in mammals and are highly diverse between species 69. Because of the absence of direct homology between mouse YM1 and human CLPs, the importance of studying murine CLPs is sometimes questioned. However, CLP family members are present in almost all mammalian species and are undergoing apparently rapid evolution. This high diversity and upregulation by IL-4 strongly suggests CLPs are involved in protection against infection, and the fundamental mechanism of action is likely to be shared across species.

AMCase and chitotriosidase, which actively degrade chitin, are thought to be important in the defense against fungal infections as well as in the degradation of inhaled chitin 68,70–72. Nance *et al*. 73 have elegantly demonstrated that AMCase activity is essential to control chitin-containing cysts in the brains of mice chronically infected with *Toxoplasma gondii*. AMCase in humans and mice is strongly associated with Th2 responses 68,70, and blockade of AMCase activity has profound consequences for Th2 mediated, allergic airway inflammation 74. It is therefore very likely that these molecules are involved in protection against Th2-inducing chitin-containing nematodes. They may also contribute to degradation of chitin-containing parasite-debris that is shed during molting. Additionally, because chitin itself has profound effects on the immune system 75, the ability to break down chitin or even just bind to it, means these proteins will be involved in shaping the immune response triggered by chitin-containing parasites.

In contrast to the active chitinases, the function of YM1 and the other CLPs is still something of a mystery. This is despite a remarkable depth of knowledge on the structural biology of these proteins, due to the propensity for YM1 to form crystals 76–78. Although crystal formation has so far only been described in the context of lung pathology, it is feasible to speculate that the large quantities of YM1 produced in the direct environment of parasites are taken up during feeding. Crystal formation could lead to internal damage in the gastrointestinal system of the parasite. This is unlikely to be sufficient for parasite killing, as both C57BL/6 and BALB/c mice produce relatively large quantities of YM1 in response to *L. sigmodontis* infection but only C57BL/6 mice are able to kill the parasites before patency 19,79. Unlike many other CLPs, YM1 does not actually bind chitin but has been shown to bind components of the extracellular matrix such as heparan sulfate 80. This would be consistent with a tissue remodeling or repair function rather than active parasite killing. YM1 has also been implicated in the immune regulation of T-cell responses by IL-4 activated dendritic cells *in vitro* and *in vivo* 81–83 and has direct effects on CD8^+^ T-cell proliferation 84. Taken together, YM1 is likely to have a role in the defense against parasitic infections but this may be through regulatory and tissue remodeling functions rather than direct anti-parasite action.

### RELMα/β

Another key molecule strongly associated with alternative macrophage activation and thus potentially important for the resistance against parasite infections is RELMα (Retnla) (also called FIZZ-1). Indeed, more than arginase or YM1, which can be induced by classical stimuli such as LPS 85,86, upregulation of RELMα is typically very reliant on the IL-4Rα for enhanced expression 13. It is important to note, however, that macrophages are not the only RELMα source. Eosinophils, neutrophils, and dendritic cells as well as non-hematopoietic cells (e.g. epithelium) express RELMα under certain circumstances 87–90. One argument for a function in host defense is that RELMβ, a closely related family member produced by epithelial cells 91, plays a direct role in the attrition and expulsion of intestinal, lumen dwelling parasites 92. This effect seems to mainly be mediated by binding of RELMβ to chemosensory organs of parasites 93 limiting worm chemotaxis and the location of their food source eventually leading to starvation and death 92. In contrast no direct interaction of RELMα with parasites or parasite products has been described so far and somewhat surprisingly RELMα has instead been associated with suppression of Th2 immune responses and reduced resistance to gastrointestinal parasite infection 59,87. Furthermore RELMα from ‘alternatively activated’ dendritic cells during the priming of a Th2 response *in vitro* leads to enhanced IL-10 production from T cells 82 and RELMα has been implicated in the induction of Th17-responses during bacterial infection 94. Thus, one of the main effects of RELMα seems to be the modulation of the adaptive immune response limiting Th2 responses and enhancing Th17- or regulatory responses. Nonetheless, RELMα may still promote parasite resistance. For example, RELMα can act as a chemoattractant for eosinophils 95,96, which are strongly connected with resistance to certain parasite infections 97.

A critical function for RELMα may prove to be in the regulation of host energy metabolism and glucose homeostasis during infection. RELMα is related to resistin, a hormone associated with insulin resistance 98 and has been linked to glucose tolerance and the regulation of serum leptin levels during colonic inflammation 90. RELMα itself has a cholesterol-lowering effect and protects against atherosclerosis in murine models 99. Although the impact of this metabolic control on helminth infection has yet to be determined, it is increasingly recognized that alternative macrophage activation not only induces a very distinct metabolic phenotype within the macrophage but also affects the metabolism of the whole organism 100–102. Thus, RELMα seems to adopt very diverse roles dependent on the context and the infection site, making its role in resistance to parasite infection difficult to dissect. Moreover, whether these differences in functionality are associated with different cellular sources or specific modifications of the RELMα protein (e.g. proteolytic cleavage, glycosylation etc.) remain to be elucidated. Overall, unlike its sibling, RELMβ, RELMα may have no direct anti-parasite effects but instead may modulate the host immune responses that affect worm survival and promote host tolerance to infection.

### Arginase

The last of the ‘Big Three’ molecules associated with alternative activation of macrophages is arginase 1 103. Arginase 1 is the enzyme that most typifies the immunomodulatory and cross-regulatory nature of macrophage activation. Arginase 1 competes with inducible nitric oxide synthase (iNOS/NOS2) for their mutual substrate L-arginine. Because iNOS is important in the production of reactive nitrogen species and microbial killing, preferential expression of arginase-1 in alternatively activated macrophages serves the dual purpose of creating important anti-nematode mediators (see below) as well as inhibiting concomitant NOS2 activity. Importantly this inhibitory effect is not restricted to just NO-production but also limits the release of pro-inflammatory cytokines by macrophages and restricts inflammatory cell recruitment during endotoxemia 104. Because arginase activity can cause systemic changes in arginine availability, the immunosuppressive effects extend beyond macrophage intrinsic effector functions and lead to impaired T cell responses 104–107. Thus like YM1 and RELMα, arginase 1 functions as a modulator of immune responsiveness. However, unlike YM1 and RELMα, arginase-1 has been directly linked to resistance to helminth infections.

Depletion of macrophages or inhibition of arginase during secondary infection with *H. polygyrus* results in loss of a protective memory response and failure to expel the parasites 28,51. Thus, although arginase expression can limit immune responses including T cell activation, it also negatively impacts on the invading parasite. Whether this is due to a reduction in the availability of L-arginine to the growing parasite remains to be tested. More recent work suggests an alternate mechanism of arginase 1-mediated parasite control. L-ornithine and polyamines, products of arginase 1 mediated arginine catabolism, reduced the migratory capacity of *H. polygyrus* larvae *in vitro* 46 and inhibition of arginase 1 prevented retention and trapping of *N. brasiliensis* larvae in the skin of infected animals 108. Proline and hydroxyproline, further downstream products of arginase activity, are central constituents of collagen 109, a major component of extracellular matrix, and are found at high levels in *S. mansoni*-induced liver granulomas and therefore potentially important in restricting larval migration. Thus, the effects of arginase on parasite survival and resistance to infection may in part be mediated through its well-known role in tissue remodeling rather than immune modulation or arginine removal. Of note, the Wynn laboratory 58 found the consequences of arginase 1 expression depend on the cellular source. In their model of liver fibrosis caused by *S. mansoni* egg deposition, macrophage-derived arginase expression was central to immunosuppressive and regulatory but not pro-fibrotic functions. They propose that fibroblast derived arginase is more important for extracellular matrix deposition 58. Taken together, these studies illustrate that both arginase expression and macrophages can be of central importance in the resistance to helminth infections, but the specific contribution of macrophage-derived arginase will need to be assessed in each model.

### INOS/ROS

As mentioned above, helminth infections are most commonly associated with a protective Th2 immune response and alternative activation of macrophages. However, anti-bacterial effector molecules such as reactive nitrogen or oxygen species due to enhanced activity of the corresponding enzymes (i.e. INOS, NOX) have been shown to effectively kill helminth parasites *in vitro* 110,111. Because this protection would come at the cost of enhanced pathology and tissue destruction 7, such responses are usually tightly controlled and suppressed. Nonetheless, several findings indicate that components of type 1 immunity do contribute to parasite killing and expulsion of helminth infections. For example, a lack of IFNγ and consequently reduced neutrophil accumulation has been associated with prolonged survival/persistence of adult *L. sigmodontis* worms in the pleural cavity of infected mice 112. Similarly formation of extracellular traps (NET), normally associated with the trapping of bacteria and fungi, have been shown to effectively kill *S. stercoralis* larvae in a collaborative effort of alternatively activated macrophages and neutrophils 55. In the case of the tapeworm *Taenia crassiceps*, type 1 responses and iNOS are essential for resistance at the early stages of infection 113. Thus, although pro-inflammatory responses are typically suppressed during helminth infections, localized expression (possibly in the context of granulomas as discussed below) with tightly managed release of cytotoxic mediators might provide additional means by which the immune system can control helminth infections. It may also be that when the type 2 effectors alone cannot effectively control infection, the contribution of more inflammatory type 1 pathways are a necessary risk, especially in the context of more lethal infections, such as those caused by some tapeworm species.

### Multinucleated giant cell formation

One characteristic of many helminth infections is the formation of dense granulomas around the invading parasite 50,114. These usually consist of highly organized layers of immune cells with macrophages forming a central part. Some of these macrophages then fuse together to form multinucleate giant cells (MNGCs). MNGCs and granulomas were originally described in the context of Th1-biased *Mycobacterium tuberculosis* infection and were thought to help prevent the spread of infectious mycobacteria 115. It is becoming more apparent, however, that granulomatous reactions are also a central part of many Th2-mediated responses, and indeed seem to be an integral part of the alternative activation program of macrophages. Many of the molecules thought to be involved in the formation of MNGC (e.g. E-cadherin, CD36, Mannose receptor) are also markers of AAMΦ, and incubation of bone marrow derived macrophages with IL-4 or IL-13 *in vitro* will lead to fusion and MNGC-formation 116–119. The consequences of macrophage fusion and granuloma formation during helminth infections are not yet clear, but the encapsulation of parasites may prevent them from causing excessive tissue damage or, as discussed above, help to trap them in tissues more easily accessible to immune cells 120. In this context, Rajan *et al*. 49 showed that macrophages attached to *B. malayi* larvae lead to deformations/changes in the underlying cuticle of the parasite which allowed eosinophils to transmigrate underneath the cuticle and potentially damage the parasite. Partial encapsulation of parasites or simple attachment of immune cells alone, however, does not seem to be sufficient to efficiently kill adult parasites as we often observe healthy looking motile filarial nematodes in which a section of the parasite is encased in a granulomatous structure. Thus entrapment might just be the first step allowing the immune system enough time to deploy additional defense mechanisms.

Attachment of macrophages to introduced foreign material (e.g. implants) creates a privileged space between the cell-membrane and the surface of the material into which degradative enzymes, acid, and reactive oxygen or nitrogen intermediates can safely be released without risk of deactivation or damage to the surrounding tissue 116,121. Macrophage fusion increases the surface area covered by the privileged space 122. MNGC-formation during nematode infection might thus constitute a form of foreign body reaction where the inability of individual macrophages to engulf a particular parasite leads to their fusion in a process called ‘frustrated phagocytosis’ 121. These fused macrophages help create a ‘restricted’ environment surrounding the parasite in which secretion of actively ‘killing’ molecules or removal of essential nutrients to starve the parasite, could limit worm survival. A major chicken and egg question remains: do parasites get encapsulated because they are dying/damaged or does the encapsulation cause their death?

### Complement

To develop both a broader and more detailed picture of the function of the IL-4Rα on macrophages during nematode infection, we recently performed an in-depth RNAseq analysis of IL-4Rα-deficient and -sufficient macrophages elicited by implantation of the peritoneal cavity with the nematode *B. malayi* or exposure to thioglycollate 9. Surprisingly, one of the most differentially regulated pathways in AAMΦ as compared to thioglycollate elicited macrophages was the complement and coagulation cascade 9. Although the upregulation of the associated transcripts was not entirely IL-4Rα dependent the sheer number of transcripts associated with this pathway exceeded all expectations. C3, the central component of all complement activation pathways, was among the most abundant gene transcripts found in nematode elicited AAMΦ. Under normal circumstances hepatocytes are the major source of C3 in serum. However, many more cell types including macrophages and monocytes can express complement factors locally 123. The functional relevance of local versus systemic complement production, however, is not well understood.

In the context of filarial nematode infection, the absence of cellular recruitment might prevent the leakage of fluids and serum components from the vasculature that would normally occur during inflammatory processes 13. Thus, local sources of these components may be required to allow efficient accumulation of complement components at the infection site, perhaps binding to the parasite and facilitating the adherence of immune cells. The contribution of complement to nematode control has been demonstrated in recent studies 46,56, and the importance of local C3-production is highlighted by the fact that parasitic nematodes have evolved counter-measures to inactivate C3 124. Of note the induction of these counter-mechanisms seems to be stage specific with early stage larvae being more susceptible than adult parasites 125. Thus, local production of complement factors by macrophages might allow rapid attack of invading parasites and avoid the induction of inflammatory processes that might lead to tissue damage. This hypothesis is also supported by an earlier finding that activated C3 acts as a chemokine attracting eosinophils but not neutrophils and thus circumventing normal inflammatory recruitment pathways 126.

It may also be that complement components are contributing to the anti-inflammatory environment of helminth infection. C1q and C3, which are both highly upregulated in AAMΦ 9, have been found to exhibit profound anti-inflammatory properties. Pretreatment with C3 containing serum enhances phagocytosis and uptake of *Francisella tularensis* by human macrophages but simultaneously reduces NF-κB signaling and pro-inflammatory cytokine secretion 127. Similarly, uptake of C1q-bound apoptotic cells by human macrophages alters LPS-induced cytokine production to a more immunoregulatory phenotype with increased release of IL-10, IL-27, and IL-33 and reduced inflammasome activation 128. Thus, similar to the many AAMΦ effector molecules, release of complement components during helminth infection might serve a dual or even multiple purposes; complement factors may allow efficient attachment of cells to invading larvae and help to prevent patent infection, while at the same time limiting excessive pro-inflammatory responses and hence pathological sequelae. Dissecting the contribution of complement to both host resistance to nematode infection, and host tolerance through suppression of inflammatory responses is an exciting area of future research.

### Metabolic re-programming and nutrient sequestration

Another very prominent characteristic evident from our RNAseq data is the dramatic change in metabolic pathways utilized by macrophages following parasite infection 9. Changes in energy metabolism have been described for IL-4 activated macrophages before 100,129 and indeed have been described as a cardinal feature of alternative activation 130. However, the extent to which these changes dominate the alternative activation phenotype have not been fully appreciated. Twenty-three of the 27 most highly regulated pathways in nematode elicited macrophages were connected to metabolic processes 9. Interestingly many of these pathways were also associated with amino acid degradation or processing (alanine, aspartate and glutamate metabolism; valine, leucine and isoleucine degradation; valine, leucine and isoleucine biosynthesis; arginine and proline metabolism; glycine serine and threonine metabolism). Given the well documented role of nutritional immunity, i.e. the withholding of essential nutrients from invading pathogens, in resistance to bacterial and intracellular infections 131,132, it is feasible that these pathways perform a similar role in helminth infection.

IL-4Rα has a major impact on the expression of specific amino acid transporters in AAMΦ. In particular, there is increased expression of transporters associated with arginine uptake (Slc7a2, Slc7a4, and Slc36a2) and downregulation of a transporter involved in arginine efflux (Slc7a8) 9. However, we do not know whether the differential regulation of these pathways provides a means of removing essential nutrients from the direct environment of the parasite and eventually leading to its starvation, or whether they provide building blocks for host metabolic processes. In this context, it is important to note that transcripts associated with uptake of metal ions (e.g. transferrin, S100a1) are also expressed at enhanced levels by AAMΦ. Thus, AAMΦ seem to increase the uptake of several essential nutrients. The metabolic changes in nematode elicited macrophages provide an exciting new avenue of investigation. Further research is required to ascertain the functional consequence for the resistance to helminth infection or indeed, tissue homeostasis during infection, as discussed below.

## Tissue repair

Macrophages are always present following tissue injury and regulate all aspects of repair, from the initial inflammatory phase, to cellular proliferation, angiogenesis, wound contraction, matrix deposition, and tissue remodeling and/or scar formation 133,134. Although the initial inflammatory phase is typically associated with M1-type macrophages, there is a large and growing literature documenting that a reprogramming of macrophages away from an M1 phenotype promotes tissue repair and regeneration (reviewed in 135). These ‘repair’ macrophages are typically called M2, but this encompasses an enormous range of potential phenotypes, and the specific contribution of IL-4Rα signaling to repair and regeneration processes still needs to be elucidated.

The evidence that IL-4Rα signaling on macrophages contributes to repair is mainly circumstantial although increasingly strong. The IL-4Rα dependent production of arginase is one of the earliest and best examples. Arginase contributes to tissue remodeling and repair because ornithine generated by arginase activity can be converted to polyamines and proline, supporting cell proliferation and collagen synthesis, respectively 109. These properties also explain the frequent association of arginase with fibrosis and in particular asthma, where it is believed to contribute to pathological tissue remodeling 136. Although it was the production of arginase by ‘M2’ macrophages that was largely responsible for them being associated with tissue repair, it seems that the pro-fibrotic activity of arginase may not be macrophage-derived. When arginase deficiency is restricted to macrophages, the T-cell suppressive but not the pro-repair/fibrosis functions are mediated by macrophages 58. Nonetheless, the ability to control inflammation is a critical component of wound repair 137 and thus macrophage-derived arginase is still likely to be an important player in many injury contexts.

In addition to arginase, RELMα and the CLP YM1 are highly but transiently upregulated in response to incisional wounding 21. Despite the reported failure to detect IL-4 or IL-13 in wounds 138, we found that the expression of these markers was entirely IL-4Ra dependent during wounding 21. There is considerable evidence for a pro-repair role for RELMα. Several papers have documented the angiogenic as well as mitogenic properties of RELMα *in vitro* 139,140. Indeed, instillation of recombinant RELMα into the lungs of mice induced a similar phenotype as seen after pneumonectomy with enhanced lung hyperplasia and epithelial cell proliferation, typical signs of wound healing 141. However, the contribution of RELMα to repair may be highly complicated by its ability to suppress Th2 responses 58,59. So although RELMα has direct repair functions, it also acts in a negative feedback loop to control fibrosis. Thus, its role in injury and wound healing is likely highly context dependent. CLPs, and in particular YM1, are frequently identified at sites of injury. However, until recently, direct evidence for involvement of CLPs in repair, was lacking. In a bleomycin model of lung injury, Zhou *et al*. 142 have now shown that Chi3l1 (BRP39) promotes repair through augmented alternative macrophage activation, fibroblast formation, and matrix deposition.

Many other repair proteins are regulated by IL-4 or IL-13 in macrophages contributing to the evidence that tissue protection is a key function for AAMΦ. For example, transcriptionally, we observed that extracellular matrix degrading matrix metalloproteases (MMPs) are actively downregulated by IL-4Rα-mediated signaling in macrophages, while their inhibitors (TIMP1 and TIMP2) are upregulated 9. Similarly, we observe IL-4Rα dependent induction of insulin-like growth factor 1 (IGF-1) during helminth infection 9 as previously demonstrated in response to Th2 cytokines *in vitro* 143. IGF-1 has a long established role in repair in part through its ability to stimulate the proliferation and survival of fibroblasts and myofibroblasts and to promote matrix production and wound closure 144,145. The importance of IGF-1 in repair during helminth infection was illustrated in a recent study by Chen *et al*. 146, in which IGF-1-producing AAMΦ were needed to prevent excessive tissue destruction and hemorrhage caused by lung migrating larvae.

Although more direct evidence is needed, there is little question that macrophages activated via the IL-4Rα contribute to repair. Wound repair, especially in adults, is often associated with scar tissue and when excessive leads to fibrosis. It has been proposed that wound healing is evolutionarily optimized for speed of healing under dirty conditions and thus scar tissue may be the price mammals must pay to close wounds sufficiently rapidly to avoid infection 147. Because it seems likely that a fundamental function of type 2 immunity is to accelerate repair, it is not surprising that AAMΦ and their products are associated with fibrosis 2. The future challenge will be to identify the specific functions of AAMΦ-derived repair molecules in specific settings and, if at all possible, to identify pro-repair versus pro-fibrotic pathways. This will require testing the quality and rate of repair in macrophage specific deletions of the IL-4Rα as well as individual effector molecules.

Because macrophages are rapidly recruited to the site of tissue injury, the predominant source of ‘wound’ macrophages are likely to be blood-circulating monocytes. The currently held view is that the early Ly6Chi monocytes are replaced by Ly6Clo ‘patrolling’ monocytes as repair progresses. Indeed, the inflammatory monocytes may only be needed to control potential infection, with the later stage macrophages more essential for full repair 133. However, with our growing understanding of the importance and longevity of tissue-resident macrophages, it seems likely that in many contexts tissue-resident macrophages will contribute, particularly at later stages of repair, when the inflammatory monocytes have gone. If so, they will need to expand in number, potentially by IL-4, or perhaps CSF-1 driven proliferation.

### Suppressing inflammation

IL-4 has long been considered an anti-inflammatory cytokine 148, and macrophages activated by IL-4 have important functions in suppressing immune responses (reviewed in 48). Inflammation needs to be controlled to allow effective wound repair 137, and thus, the anti-inflammatory nature of AAMΦ is intimately linked to their wound repair phenotype. This makes evolutionary sense in the context of the adaptive type 2 response: a host infected with macroparasites would want to repair any damage caused by a large tissue migrating parasite but also avoid the serious collateral consequences of mounting an inflammatory response to the pathogen.

The data supporting an anti-inflammatory role for AAMΦ have come from many laboratories and has been mostly based on the evidence that AAMΦ are important sources of immunosuppressive cytokines. These include TGF-β 149,150, PGE2 151, and the IL-1 receptor antagonist 149,152. The chemokine expression profile is also strongly associated with a non-inflammatory role 153 indicated by specific downregulation of key pro-inflammatory chemokines via IL-4 8,9,152.

The ability of a single protein to be both anti-inflammatory and a central mediator of repair is demonstrated by TGF-β, which is known for both immune suppression and fibrosis 154. This functional duality is typical of many Th2-activated macrophage products. As discussed earlier, Arg-1 promotes collagen deposition on one hand but inhibits nitric oxide production and T-cell activation on the other. 12/15 lipoxygenase, a protein strongly associated with IL-4 activation of macrophages in both mice and people, is needed for effective wound repair 155 but attenuates pro-inflammatory macrophage activation 156. RELMα exhibits angiogenic properties 139 and YM1/2 bind extracellular matrix 157, but these are also implicated in regulating inflammation 59,81,87. Similarly, Chi3l1 contributed to repair in a model of bleomycin-induced lung injury both through direct repair functions and suppression of inflammation 142. Thus, modulation of inflammation is intrinsically linked to wound repair and an important characteristic of AAMΦ.

Although IL-10 is very strongly associated with a M2 phenotype and is often listed as a prototypic cytokine associated with alternative activation 47,149, new sequencing technologies and tools for assessing cell-specific expression *in vivo*, have made the story more complicated. Despite abundant expression of the IL-10 receptor, YM1, RELMα, arginase and many other AA markers, RNAseq analysis of nematode-elicited macrophages had sufficient depth of coverage to be able to say that IL-10 is not produced by F4/80hi macrophages in this context 9. The important source of IL-10 following both hookworm migration through the lung and filarial nematode infection appears to be T cells 146,158. This is consistent with recent data showing anti-inflammatory functions of gut macrophages are mediated not by their ability to produce IL-10 but by IL-10 receptor expression 159. The lack of IL-10 production by AAMΦ may be surprising considering the known ability of macrophages to produce IL-10 in response to antibody cross-linking 160, and indeed we would expect antibodies to be present in the helminth models discussed above. However, IL-10 production by macrophages may require additional signals, such as TLR ligands and type 1 interferons 161,162, that may not be present in these nematode infection sites. The absence of macrophage derived IL-10 despite high levels of parasite specific antibody and antigen, may be further explained by Fcγ receptor usage on AAMΦ during helminth infection. We found that IL-4Rα signaling drives a switch from the activating Fc receptors involved in IL-10 production 160 to the inhibitory FcγrIIb 9,163.

The combined anti-inflammatory/wound healing function of AAMΦ is strongly supported by a study of *S. mansoni* infection in mice that lack the IL-4Rα specifically on macrophages and neutrophils but have otherwise intact Th2 responses 60. Following *S. mansoni* infection, these mice died from overwhelming inflammatory responses in the intestine and leakage of bacteria into the blood. Although not conclusive evidence, the data strongly suggest that in the absence of AAMΦ, these mice were unable to repair the damage caused by egg migration through the intestinal wall and the subsequent septic inflammation 60. The specific roles Th2-induced proteins play in the complex orchestra of tissue repair and remodeling are still to be established, but a rapid shutdown of the inflammatory response to injury is likely an important contribution.

## Immune regulation

**Fig 4:**
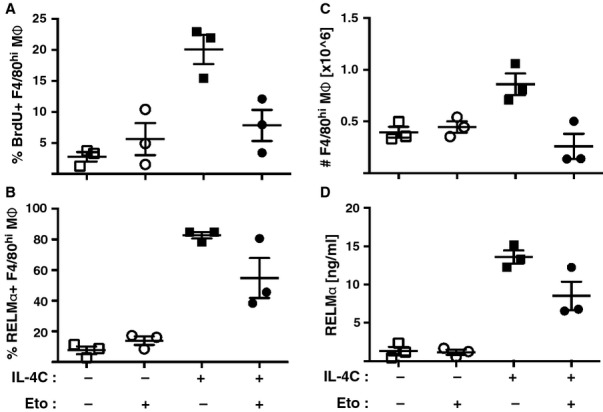
Inhibition of fatty acid oxidation blocks macrophage alternative activation and proliferation *in vivo*. C57BL/6 mice were injected i.p. with 0.5 mg Etomoxir (Eto, Sigma Aldrich) to inhibit fatty acid oxidation 1 h prior to treatment with IL-4C. Detailed description of similar experiments can be found in Ruckerl *et al*. 67. (A) 24 h later, the proliferative expansion of F4/80^high^ cells was determined by 3hr-BrdU pulse and flow cytometry. Data points indicate individual animals, and lines indicate mean. (B) Intracellular RELMα expression in the cells analyzed in (A). (C) Number of F4/80^high^ cells present in the pleural cavity of the mice analyzed in (A). (D) Concentration of RELMα in the lavage fluid isolated from the pleural cavity of the mice analyzed in (A).

A paradox that still needs to be resolved is that AAMΦ, which actively proliferate *in vivo*, are profoundly antiproliferative *ex vivo*. AAMΦ suppress proliferation of cells with which they are co-cultured *ex vivo* in an IL-4-dependent manner 150,164. This property was our first insight into the distinct function of macrophages under IL-4Rα control and has been reproduced in many laboratories where suppression has been attributed to arginase depletion 58, TGF-β 165, PD-L pathway 166,167, and 12/15 lipoxygenase 168. Of note, rapid division of immune cells (i.e. effector T cells) has previously been shown to be highly reliant on glucose and the Warburg effect 169. In contrast, alternative activation and also IL-4-driven proliferation of macrophages are dependent on fatty acid oxidation and oxidative phosphorylation (OXPHOS) 100 (*Fig. *4). The reasons for these metabolic differences are not yet completely clear. Studies in T cells suggest that aerobic glycolysis allows for quick accumulation of biomass needed for cellular expansion whereas OXPHOS is associated with the enhanced lifespan of memory T cells 170,171. In an analogous manner, the utilization of OXPHOS in AAMΦ might impart slightly slower proliferative expansion but allow the expanded population to survive for a longer period of time. Another hypothesis is that metabolic differences are linked to the anatomical location in which cellular expansion is likely to occur. T cells normally expand within lymphoid structures which are surrounded by adipose tissue and well vascularized 172,173. Thus, it is feasible to assume that these structures provide ample nutrients for optimal, rapid cellular expansion. In contrast AAMΦ expand in the tissues in response to IL-4 13. In the presence of a large, tissue-destructive macroparasite and the accompanying wound healing process, these might suffer from acute shortage of essential nutrients, especially glucose. Thus, the adoption of OXPHOS as a means of generating energy might be necessary to allow the expansion of tissue-resident macrophages during helminth infection. In this context, it is interesting to note that certain tumor cell lines, normally well known for their high glucose consumption and glycolytic energy metabolism, adopt OXPHOS to maintain cellular proliferation under low glucose conditions 174. Furthermore, we observe a highly significant increase in expression of the arginine transporter CAT2 (Slc7a2), as well as other amino acid and nutrient transporters in response to IL-4Rα signaling in AAMΦ 9. Transporter upregulation, in addition to the above-discussed effector functions, might allow macrophages to acquire essential nutrients needed for expansion. Thus, the anti-proliferative effect of AAMΦ on bystander cells *in vitro* might at least in part be a side effect of nutrient sequestration to which T cells are highly sensitive 175.

Critically, whether AAMΦ directly affect T-cell proliferation and polarization *in vivo* is not known. Although individual molecules expressed by AAMΦ (i.e. RELMα, YM1, arginase) have been associated with immune suppression 58,59,87, it is not always clear whether it is expression by macrophages that imparts this effect. Indeed, our RNAseq data indicate a remarkable lack of chemokine receptor expression on nematode elicited macrophages 9, suggesting that these cells are prone to stay in the tissues and most likely do not migrate to the draining lymph nodes to direct immune response. Thus, the immune regulation observed *in vivo* 58,59,87 might rather be due to other cells, such as DCs 82, expressing AAMΦ-associated molecules or due to alternative activation of macrophages resident within the lymphoid structures 79. Undoubtedly AAMΦ do influence the local immune milieu in the tissues. Recruitment of eosinophils to the site of helminth infection is greatly enhanced by the presence of AAMΦ 176 and recruitment of monocytes and neutrophils is discouraged by IL-4 9,19. Thus, AAMΦ contribute to regulating cells in their immediate vicinity via various means and although it has yet to be documented, local T-cell proliferation may also be prevented. As described above in the context of complement, a major feature of AAMΦ may be to regulate the local immune environment and mediate key necessary effector functions, leaving more systemic immune regulation to other cells. In this context, the local expansion of the resident population and avoidance of pro-inflammatory recruitment makes sense; shaping a ‘safe’ environment for the control of helminth infection or to mediate repair.

## Plasticity

Classical macrophage activation, as found during bacterial infections, and IL-4Rα-mediated alternative activation are two extremes of a wide spectrum of overlapping activation phenotypes 160. Although differentially activating stimuli will block or reduce opposing activation signals, macrophages are known to be highly plastic and rarely exhibit fixed phenotypes. Instead they form a continuum, adopting characteristics of activation as needed according to their environment 177. The lack of a fixed activation state is supported by data showing that once the initial stimulating agent is removed, macrophages quickly lose their activation phenotype (178, own unpublished data) and cells isolated from one activating environment subjected to an opposing stimulus will adopt characteristics of the newly activating environment 179. Indeed, macrophages isolated from *B. malayi* infected animals and subjected to stimulation with LPS/IFNγ readily express pro-inflammatory cytokines (e.g. IL-6, IL-10, TNF) and are able to control intracellular replication of *Leishmania mexicana* parasites 85. This flexibility in activation phenotype, demonstrated *in vitro*, is presumed to be important in the context of co-infections allowing macrophages to quickly adapt to new challenges.

Changes in macrophage activation phenotype *in vivo* have been demonstrated in the setting of wound healing, where initially pro-inflammatory macrophages eventually convert to a more anti-inflammatory phenotype 138. Conversely we have reported a change in cytokine expression by macrophages isolated from *B. malayi* infected mice early or late following parasite implantation, with an increase in pro-inflammatory cytokine production at later stages 180. However, *in vivo* macrophage activation, especially in the context of infection, is rarely homogenous, and only a subset of macrophages will show signs of activation 13,32,181. Thus, adaptation to environmental changes could at least in part be due to induction of differentially activated subsets of macrophages, rather than true plasticity. Moreover, waves of cellular recruitment/expansion of various macrophage populations might be favored over re-polarization of the existing already activated population. Thus, despite clear evidence of macrophage plasticity *in vitro*, macrophage plasticity during infection has to be re-evaluated. This is especially true in light of our new understanding of macrophage origins and proliferation 24 and the findings by Gundra *et al*. 20. Macrophages derived from different cellular sources (i.e. blood monocytes versus tissue-resident macrophages) respond differently to the same stimulus (i.e. IL-4) with potentially different functional activation outcomes. Of note, functional differences between resident and monocyte-derived macrophages have been described previously. Uderhardt *et al*. 182 showed preferential uptake of apoptotic cells by resident peritoneal macrophages over inflammatory monocyte-derived cells. Disruption of this bias through genetic ablation of 12/15 lipoxygenase resulted in presentation and recognition of self-antigens leading to autoimmune reactions and lupus-like symptoms 182. Furthermore, although *B. malayi* derived AAMΦ readily upregulated iNOS and pro-inflammatory cytokine-production following stimulation with LPS/IFNγ, we were never able to detect release of IL-12 from these cells, normally a key feature of classical macrophage activation 85. Thus, it is likely that epigenetic or otherwise mediated determinants of macrophage phenotype are induced during development restricting the plasticity/variation in responses 178,183.

Nonetheless, bone marrow-derived macrophages can adopt a resident-like phenotype and re-populate the tissues after lethal irradiation 24. Thus, these developmental restrictions may be imposed by the local environment rather than in the bone marrow or during embryogenesis. Local regulation of macrophage identity was highlighted in two recent studies, in which expression of the transcription factor GATA6 was shown to be essential for the resident peritoneal macrophage phenotype 184,185. Furthermore, all-trans retinoic acid and omentum-derived soluble factors could drive expression of resident peritoneal macrophage signature genes in bone marrow derived macrophages *in vitro* 184. Thus, the environment of the peritoneal cavity seems necessary and sufficient to induce the resident phenotype. Although this does not occur in monocyte derived macrophages present in the peritoneal cavity under normal circumstances it highlights the context dependency and flexibility of macrophage phenotypes. In this context, the macrophages recruited to the site of *B. malayi* implant, as analyzed in our RNAseq analysis, are monocytic in origin (authors’ unpublished data). Despite clear evidence of proliferation and adoption of a resident-like phenotype (i.e. F4/80^high^, MHC-II ^low^) 13, data from protected irradiated chimeras suggest that an initial inflammatory influx, caused by the wounding of the surgical incision, leads to the accumulation of monocyte derived cells which are then converted to a resident like phenotype. Thus, in addition to driving proliferative expansion of resident and monocyte-derived macrophages, IL-4 seems to enhance or enable the conversion of monocyte derived macrophages to a resident phenotype. Consistent with this hypothesis, macrophages from *B. malayi* implanted animals showed highly enhanced GATA6 expression as compared to thioglycollate elicited macrophages 9, and IL-4 induces GATA-6 expression on thioglycollate-elicited macrophages 20. The functional consequences of cellular source, proliferative expansion, and long-term residence of tissue macrophages on the resistance to helminth infection remain a wide open question.

## The future

We are entering a new era in macrophage biology, particularly in light of our new understanding of cellular origins. New questions are emerging daily, and we have raised many of them here in this review. For example, in the context of helminth infection, how do single molecules perform such diverse functions as parasite killing, immune suppression, and wound repair? More broadly, what is the extent of macrophage plasticity? Do recruited cells truly become fully functional resident cells? What are the epigenetic/transcriptional controls that regulate these functions both during development and locally in the tissues? New tools 186,187 and collaborations with different disciplines from stem cell biology to systems biology will allow us to unravel some of the enormous complexity underlying macrophage function in health and disease. Studying these intricate dynamics in the context of infection with helminths, our ancient evolutionary partners, will further enhance our fundamental understanding of macrophage biology. The challenge will be to translate this new knowledge into the ability to fine tune responses to the benefit of infected or injured hosts.
